# Severe Hypernatremic Dehydration in an Exclusively Breastfed Neonate

**DOI:** 10.7759/cureus.102947

**Published:** 2026-02-04

**Authors:** Marta Figueiredo, Melissa Figueiredo, Filipa Vieira, Mónica Marçal, Madalena Tuna

**Affiliations:** 1 Neonatology, Hospital de São Francisco Xavier, Lisbon, PRT; 2 Nova Medical School, Universidade Nova de Lisboa, Lisbon, PRT

**Keywords:** breastfeeding, dehydration, hypernatremia, neonate, neurological sequelae, weight loss

## Abstract

Neonatal hypernatremic dehydration is a serious condition associated with inadequate fluid intake, often due to insufficient lactation. In addition to the complications directly related to the condition itself, the re-establishment of plasma tonicity may also lead to serious consequences if done abruptly. Management involves careful fluid restoration and gradual correction of electrolyte imbalance. The increasing incidence of this clinical condition highlights the importance of raising awareness among parents and healthcare professionals for early diagnosis and prevention. We report a case of severe hypernatremic dehydration in a 16-day-old exclusively breastfed newborn, emphasizing its clinical management.

## Introduction

Exclusive breastfeeding is the gold standard in infant nutrition. The World Health Organization recommends exclusive breastfeeding for the first six months of life [1-3], as it reduces the incidence of acute infections, lowers the risk of chronic diseases, and supports optimal neurodevelopment [1]. During this period, breast milk alone is sufficient to meet all nutritional needs [4], and it should remain an essential part of the infant’s diet until at least two years of age [2,3]. Ensuring adequate breastfeeding can be challenging during the early postpartum period. Successful breastfeeding depends on several factors, including sufficient milk production and effective milk transfer, which are determined by appropriate positioning and suction of the neonate, maternal confidence, and the frequency and duration of feedings [4]. Despite its well-established benefits, insufficient breastfeeding can lead to severe weight loss and hypernatremic dehydration, bearing neonatal morbidity and mortality [3,5]. Although most healthy neonates can be exclusively breastfed without the need for supplementation, early identification of those at risk of hypernatremic dehydration is crucial, as they may require timely and appropriate supplementation [1]. The rising incidence of hypernatremic dehydration reinforces the need for increased awareness and a high index of suspicion for diagnosing this potentially life-threatening condition [3].

## Case presentation

A 16-day-old female neonate was admitted to our neonatal intensive care unit (NICU) due to weight loss and severe dehydration. She was born at 40 weeks of gestation to a healthy 34-year-old mother, tertigravida, who had exclusively breastfed her previous children until two months of age. The pregnancy was uneventful, and there was no history of consanguinity, maternal illnesses, or obstetric complications. The infant was born via spontaneous vaginal delivery with Apgar scores of 8 and 10. Birth weight was 4190 g (90th-97th percentile), with a length of 48.5 cm (10th-50th percentile) and a head circumference of 36.5 cm (90th percentile). She was discharged home on the second day of life, exclusively breastfed, with a 10% weight loss from birth weight and in good general condition, with an unremarkable physical examination and preserved urine and stool output. At the primary care follow-up visit on day eight of life, she continued to appear clinically well, with hydrated mucous membranes, normotensive fontanelles, and age-appropriate urine and stool output; however, her weight had fallen to 3320 g, corresponding to a 21% reduction from birth weight. The mother was advised to increase breastfeeding frequency and return for weekly reassessment. On day 16 of life, she weighed 2700 g, reflecting a 35.5% weight loss compared with her birth weight. The neonate was transferred to our pediatric emergency department. There was no history of fever, vomiting, or diarrhea, but the mother reported decreased bowel movements and reduced urine output during the previous three days.

**Figure 1:**
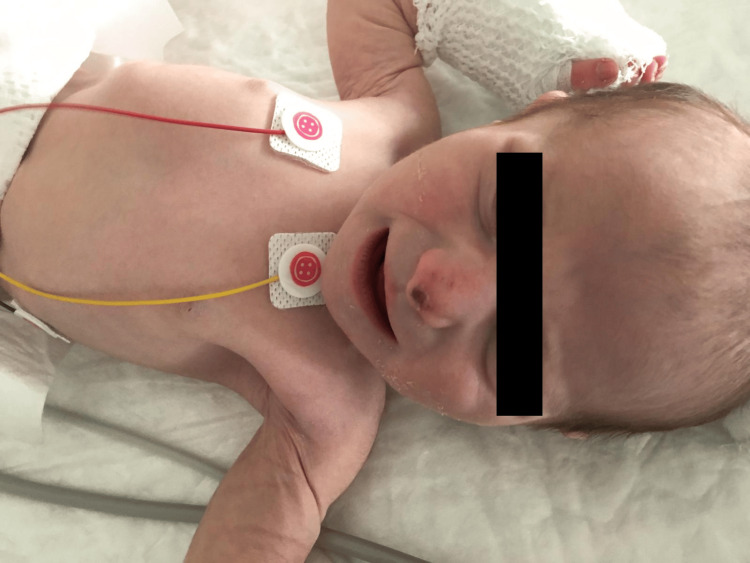
Neonate at admission, with sunken eyes, dry mouth, and poor skin turgor

**Figure 2:**
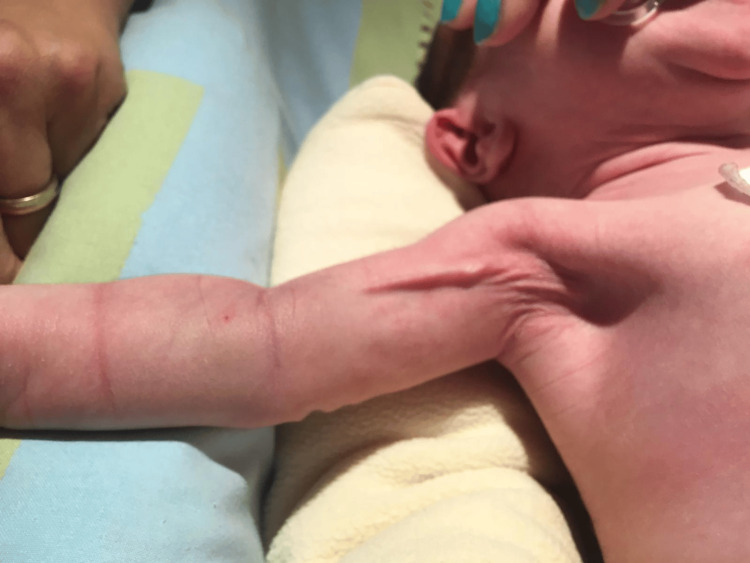
Neonate's poor skin turgor

On admission, the newborn was lethargic but irritable when stimulated and presented signs of severe dehydration (Figures 1, 2), including sunken eyes, dry mucous membranes, absence of tears when crying, poor skin turgor, and overlapping cranial sutures.

Vital signs included a blood pressure of 79/53 mmHg, heart rate of 115 beats per minute, respiratory rate of 57 breaths per minute, and temperature of 37ºC. Neurological examination revealed weak primitive reflexes and minimal responses to sound and light. The remaining systemic examination was unremarkable. Laboratory tests on admission (Table 1) revealed severe hypernatremia, acute kidney injury with pre-renal azotemia, hyperglycemia, and metabolic acidosis.

**Table 1:** Laboratory workup on admission HCO₃⁻, bicarbonate; pCO₂, partial pressure of carbon dioxide.

Parameter	Value	Reference range
Hemoglobin (g/dL)	20.9	12.5-20.5
Leukocytes (/L)	31 x 10^9^	7.0-23.0 x 10^9^
Neutrophils (%)	11.4 x 10^9^	1.5-9.5 x10^9^
Platelets (/L)	282 x 10^9^	210-650 x10^9^
C-reactive protein (mg/dL)	<0.1	<0.5
Serum glucose (mg/dL)	215.0	74.0-106.0
Creatinine (mg/dL)	4.0	0.24-0.85
Blood urea (mg/dL)	313.0	9.0-41.0
Sodium (mmol/L)	191.0	136.0-145.0
Potassium (mmol/L)	5.7	3.5-5.1
Total bilirubin (mg/dL)	2.2	<1
Venous pH	7.3	7.35-7.45
pCO_2_ (mmHg)	28.0	35.0-45.0
Base excess (mmol/L)	-12.6	<5.0
HCO_3_^-^ (mmol/L)	16.1	21.0-28.0
Lactate (mmol/L)	3.7	0.5-2.0

Fluid therapy was initiated according to our NICU protocol for the management of neonatal hypernatremic dehydration with serum sodium levels above 160 mmol/L, as detailed in the Discussion section. As the newborn was hemodynamically stable at presentation, no fluid boluses were required, and rehydration was initiated with intravenous fluids according to the correction phase of the protocol.

**Figure 3:**
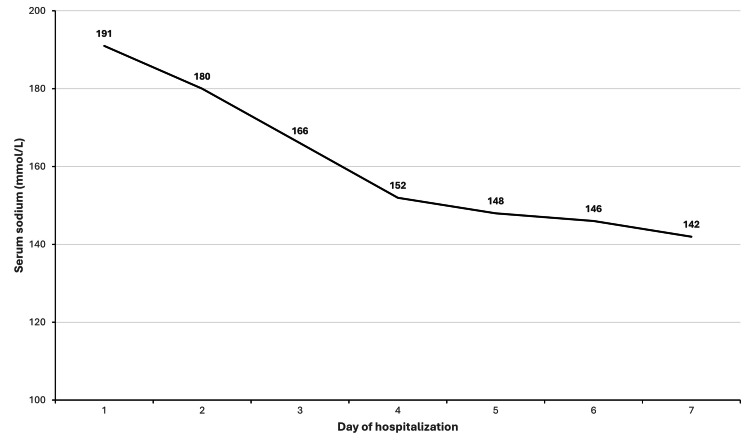
Decrease in natremia during hospital stay

Blood urea and creatinine normalized by day five of admission and the natremia by day six, with a controlled sodium reduction of 0.4 mmol/L per hour over the first 96 hours of hospitalization (Figure 3). 

Throughout admission, the patient remained hemodynamically stable and spontaneously breathing. Continuous monitoring with amplitude-integrated electroencephalography and near-infrared spectroscopy from days one to five of admission revealed no abnormal events. Serial cranial ultrasounds revealed mineralizing vasculopathy of the lenticulostriate arteries and areas of increased periventricular echogenicity in the white matter, with progressive resolution. Findings were confirmed by cranial magnetic resonance imaging (MRI) on the 15th day of admission, with no other alterations. Trophic feeding was initiated on the second day, but advancement was cautious due to feeding intolerance in the first days. Hyperglycemia was resolved by the fourth day, and full enteral feeding was achieved on the eleventh day. She was discharged on the 19th day of hospitalization, weighing 3860 g, with a normal neurological examination, tolerating both breastfeeding and formula feeding. Subsequent otorhinolaryngology evaluation showed no evidence of hearing impairment. The patient remained under regular pediatric follow-up and, at six years of age, demonstrates appropriate growth and psychomotor development.

## Discussion

Hypernatremic dehydration in neonates is a potentially life-threatening condition, defined by a sodium level exceeding 145 mEq/L. It is classified as mild (145-149 mEq/L), moderate (150-160 mEq/L), or severe (>160 mEq/L) hypernatremia [1].

The main cause of breastfeeding-related hypernatremia is inadequate fluid intake due to lactation failure, particularly in exclusively breastfed neonates during the first days to weeks of life [1,3,4]. This can result from low maternal milk supply, ineffective milk transfer, or a combination of both [1,6]. Insufficient milk supply can be attributed to low colostrum volume, delayed lactogenesis, or inadequate mature milk supply [1]. Reduced breastfeeding frequency has also been associated with higher sodium levels in breast milk [4].

Although once considered rare, the incidence of hypernatremic dehydration has been rising in recent years [1,7], coinciding with growing efforts to promote exclusive breastfeeding before hospital discharge [1,6]. Determining its precise prevalence remains challenging due to geographic variation, limited post-discharge follow-up data, and a lack of universally adopted screening protocols for neonatal hypernatremia [1,6,8]. Studies suggest that hypernatremia in healthy, term, exclusively breastfed newborns is more common than previously estimated, and that a 10% weight loss threshold, often used as a risk indicator, fails to predict many cases of mild-to-moderate hypernatremia [1].

Identified risk factors for hypernatremic dehydration include maternal obesity, cesarian section delivery, primiparity, breast-related issues, low maternal knowledge about breastfeeding, lack of previous breastfeeding experience, inadequate breastfeeding technique, and delayed initiation of breastfeeding [1,4]. Early hospital discharge without adequate lactation support and the absence of timely follow-up visits are also major contributors [1,5,8,9].

Neonates with hypernatremic dehydration may present with early signs of poor feeding, such as high-pitched, inconsolable crying, frequent feeding (less than two-hour intervals), or prolonged feeding sessions [1]. However, some may show few signs of distress, appearing satisfied after breastfeeding and often failing to wake spontaneously for feeds, delaying the diagnosis [1,9]. Other manifestations include excessive weight loss, delayed capillary refill, poor skin turgor, sunken fontanelles, lethargy, irritability, reduced urine and stool output, hyperbilirubinemia, hyperthermia or hypothermia, hypoglycemia or hyperglycemia, metabolic acidosis, and bradycardia [1,3,4,7-9]. A compensatory water shift from the intracellular to the extracellular compartment may preserve skin turgor in the early stages, masking the severity of dehydration [1,9,10]. Laboratory evaluation is essential for diagnosis and assessment of systemic repercussions. Recommended tests include complete blood count, C-reactive protein, blood glucose, urea, creatinine, electrolytes, osmolality, calcium, phosphorus, and blood gas analysis [7,8]. Early recognition of at-risk neonates and a high index of suspicion are critical, as hypernatremic dehydration can result in severe complications, including seizures, intracranial hemorrhage, cerebral edema or infarction, acute kidney injury, liver failure, venous and arterial thrombosis, disseminated intravascular coagulation, shock, and death [1,4,8]. Long-term sequelae are common, such as intellectual disability and epilepsy, highlighting the importance of structured neurodevelopmental follow-up [1,4]. Prognosis depends on the severity of hypernatremia, the timing of intervention, and the rate of sodium correction [7]. Higher serum sodium levels are associated with increased risk of treatment-related complications [6].

The rate of hypernatremia correction depends on the speed of its onset. Except in cases of acute massive sodium overload, rehydration should be gradual, with the goal of reducing serum sodium by no more than 0.5 mEq/L per hour or 12 mEq/day over at least 48 hours. This cautious approach prevents rapid osmotic shifts that may cause cerebral edema [1,6,7,10]. The duration of correction depends on the initial sodium concentration and requires close monitoring to avoid overly rapid reductions [8,10]. Clinically stable neonates with mild hypernatremic dehydration (serum sodium <150 mEq/L) may be managed with oral hydration at 100 mL/kg/day, with close monitoring of serum sodium, whereas moderate to severe hypernatremic dehydration or clinical instability requires intravenous hydration [7].

Management of neonatal hypernatremic dehydration requires careful fluid therapy aimed at restoring intravascular volume and correcting serum sodium gradually. In neonates presenting with hypovolemic shock, initial resuscitation should be performed with 10 mL/kg of 0.9% sodium chloride administered over 20 minutes. Once clinical stability is achieved, the correction phase should be initiated to avoid abrupt intravascular volume expansion. If a second bolus is required, the sodium concentration of the infused solution should be 10-15 mmol/L lower than the patient’s serum sodium level. The occurrence of tonic-clonic seizures during or immediately after resuscitation suggests cerebral edema secondary to a rapid decline in serum sodium, in which case a 3 mL/kg bolus of 3% sodium chloride is indicated.

In hemodynamically stable neonates with serum sodium levels above 160 mmol/L, the first step is to calculate the free water deficit (in milliliters) using the following formula: 0.7 × birth weight (kg) × ((serum sodium/145) - 1). The choice of rehydration fluid depends on the degree of hypernatremia: when serum sodium is ≤170 mmol/L, 0.45% sodium chloride with 5% dextrose is recommended; when serum sodium exceeds 170 mmol/L, correction should be initiated with 0.9% sodium chloride with 5% dextrose, switching to 0.45% sodium chloride with 5% dextrose as soon as possible, according to natremia. The correction rate must be carefully calculated to ensure a gradual reduction in sodium, not exceeding 0.5 mmol/L per hour. For values above 170 mmol/L, the correction time can be estimated using the following formula: (serum sodium - 145)/0.5. The infusion rate is determined by dividing the calculated water deficit by the number of correction hours. Maintenance fluids should be prescribed at 100 mL/kg/day based on birth weight, using the same solution selected for correction (0.9% sodium chloride with 5% dextrose or 0.45% sodium chloride with 5% dextrose), with the addition of amino acids, potassium, or calcium as clinically indicated. Whenever possible, the sodium content of the infused fluids should be progressively reduced. The combined volume of maintenance and correction fluids should not exceed twice the calculated maintenance requirement over 24 hours, and both infusions should be administered separately to allow independent adjustments. Serial monitoring of electrolytes every 1-2 hours is recommended until clinical stabilization. Enteral feeding may be cautiously initiated once serum sodium has decreased below 160 mmol/L and the neonate is clinically stable.

Infants with hypernatremic dehydration should undergo an electroencephalogram, cranial ultrasound, and MRI during hospitalization, given the high risk of brain injury and seizure activity. Abnormal findings may help guide early neurodevelopmental interventions and antiepileptic therapy [1].

In the present case, sodium correction was closely monitored and remained within recommended safety thresholds overall. Although minor variations occurred during the correction phase, no clinical or electroencephalographic evidence of cerebral edema was observed, and the neurological outcome was favorable. This supports previous observations that carefully controlled correction of severe hypernatremia can result in good neurological recovery [4,7].

Given the serious risks of hypernatremic dehydration, prevention is essential both during hospital stay and after discharge. Key strategies include serial assessment of breast milk supply and feeding effectiveness, as well as regular monitoring of weight loss. Sodium levels should be measured in neonates with significant weight loss or clinical signs of poor feeding, and formula supplementation should be considered when indicated. Parental education on breastfeeding techniques and warning signs of dehydration is also critical [1].

This case illustrates the importance of these measures. The newborn was discharged on day two of life, exclusively breastfed, already with a 10% weight loss. At the first follow-up visit on day eight, despite a 21% weight loss, the mother was advised to continue exclusive breastfeeding without further intervention. This highlights the urgent need for improved education on neonatal hypernatremic dehydration, not only for parents but also for healthcare providers. Our case findings are consistent with the existing literature regarding the risk factors, clinical presentation, and management of neonatal hypernatremic dehydration. Similar to previously published reports, our patient was exclusively breastfed, discharged early, and not adequately monitored [1,4,6,8], which contributed to delayed recognition and severe weight loss. Despite severe hypernatremia, the neurological findings were mild, aligning with studies suggesting that timely and carefully controlled correction of sodium can lead to favorable outcomes [4,7].

Our case stands out in the literature by highlighting severe weight loss and hypernatremia, with full recovery without sequelae, which is rarely documented, and by illustrating the importance of combining structured lactation support with systematic follow-up to prevent severe complications. These findings reinforce the current evidence, emphasizing that prevention, early diagnosis, and careful management of fluid therapy are essential to improving prognosis.

## Conclusions

Neonatal hypernatremic dehydration is a potentially life-threatening condition, particularly in exclusively breastfed neonates, and is more common than previously believed. Its management presents a significant clinical challenge, as slow and carefully controlled correction of serum sodium is essential to prevent neurological sequelae. Early identification of at-risk neonates is crucial for timely diagnosis and intervention, improving overall prognosis. Frequent and effective breastfeeding plays a key role in maintaining adequate hydration and preventing hypernatremic dehydration. Breastfeeding counseling and technique optimization should be reinforced before hospital discharge and regularly reassessed at follow-up visits. A comprehensive prevention strategy must include education for both parents and healthcare providers on abnormal feeding behaviors, excessive weight loss, and warning signs of dehydration, particularly in the first weeks of life. Furthermore, neonates with signs of inadequate feeding should undergo serum sodium screening to allow early intervention and avoid serious complications.

## References

[1] Del Castillo-Hegyi C, Achilles J, Segrave-Daly BJ, Hafken L (2022). Fatal hypernatremic dehydration in a term exclusively breastfed newborn. Children (Basel).

[2] Mosca F, Giannì ML (2017). Human milk: composition and health benefits. Pediatr Med Chir.

[3] Ünver Korğalı E, Cihan MK, Oğuzalp T, Şahinbaş A, Ekici M (2017). Hypernatremic dehydration in breastfed term infants: retrospective evaluation of 159 cases. Breastfeed Med.

[4] Arora I, Juneja H, Bhandekar H, Chandankhede M (2024). Neonatal hypernatremic dehydration in breastfed neonates: a prospective study unmasking the influences of breastfeeding practices and early weight monitoring. J Matern Fetal Neonatal Med.

[5] Shivaprakash NC, Ahmed T (2015). Hypernatremic dehydration in newborns. Int J Contemp Pediatr.

[6] Bischoff AR, Dornelles AD, Carvalho CG (2017). Treatment of hypernatremia in breastfeeding neonates: a systematic review. Biomed Hub.

[7] Ashraf M, Qureshi UA, Bhat NA (2022). Neonatal hypernatremic dehydration. Asian J Pediatr Nephrol.

[8] Durrani NU, Imam AA, Soni N (2022). Hypernatremia in newborns: a practical approach to management. Biomed Hub.

[9] Ogbe Z, Andegiorgish AK, Zeray AH, Zeng L (2020). Neonatal hypernatremic dehydration associated with lactation failure. Case Rep Crit Care.

[10] Mirnia K, Saeedi M, Ghadamghahi F (2025). Practical management of neonatal hypernatremic dehydration: a clinical study. Nephro-Urol Mon.

